# Feasibility trial evaluation of a physical activity and screen-viewing course for parents of 6 to 8 year-old children: Teamplay

**DOI:** 10.1186/1479-5868-10-31

**Published:** 2013-03-04

**Authors:** Russell Jago, Simon J Sebire, Katrina M Turner, Georgina F Bentley, Joanna K Goodred, Kenneth R Fox, Sarah Stewart-Brown, Patricia J Lucas

**Affiliations:** 1 Centre for Exercise, Nutrition & Health Sciences, School for Policy Studies, University of Bristol, Bristol, UK; 2 School of Social and Community Medicine, University of Bristol, Bristol, UK; 3 Warwick Medical School, University of Warwick, Coventry, UK; 4 Centre for Research in Health and Social Care, School for Policy Studies, University of Bristol, Bristol, UK

**Keywords:** Parenting, Intervention, Feasibility trial, Physical activity, TV

## Abstract

**Background:**

Many children spend too much time screen-viewing (watching TV, surfing the internet and playing video games) and do not meet physical activity (PA) guidelines. Parents are important influences on children’s PA and screen-viewing (SV). There is a shortage of parent-focused interventions to change children’s PA and SV.

**Methods:**

Teamplay was a two arm individualized randomized controlled feasibility trial. Participants were parents of 6–8 year old children. Intervention participants were invited to attend an eight week parenting program with each session lasting 2 hours. Children and parents wore an accelerometer for seven days and minutes of moderate-to-vigorous intensity PA (MVPA) were derived. Parents were also asked to report the average number of hours per day that both they and the target child spent watching TV. Measures were assessed at baseline (time 0) at the end of the intervention (week 8) and 2 months after the intervention had ended (week 16).

**Results:**

There were 75 participants who provided consent and were randomized but 27 participants withdrew post-randomization. Children in the intervention group engaged in 2.6 fewer minutes of weekday MVPA at Time 1 but engaged in 11 more minutes of weekend MVPA. At Time 1 the intervention parents engaged in 9 more minutes of weekday MVPA and 13 more minutes of weekend MVPA. The proportion of children in the intervention group watching ≥ 2 hours per day of TV on weekend days decreased after the intervention (time 0 = 76%, time 1 = 39%, time 2 = 50%), while the control group proportion increased slightly (79%, 86% and 87%). Parental weekday TV watching decreased in both groups. In post-study interviews many mothers reported problems associated with wearing the accelerometers. In terms of a future full-scale trial, a sample of between 80 and 340 families would be needed to detect a mean difference of 10-minutes of weekend MVPA.

**Conclusions:**

Teamplay is a promising parenting program in an under-researched area. The intervention was acceptable to parents, and all elements of the study protocol were successfully completed. Simple changes to the trial protocol could result in more complete data collection and study engagement.

## Background

Physical activity (PA) is associated with lower levels of many risk factors among children including lipid levels [1,2], blood pressure [1,2] and body mass [3,4]. Screen-viewing (SV), (e.g., watching television, playing video games and surfing the internet), is associated with an increased risk of heart disease [5] and obesity [3]. Many children exceed recommended hours of SV [6-8] and do not engage in sufficient amounts of PA [9,10]. The early school years (6–8 years of age) are a key period when children’s PA and SV behaviors are established [3]. A number of systematic reviews have indicated that currently there are only a limited number of effective interventions to change children’s physical activity [11] or prevent obesity via increased PA [12-15] highlighting a need for new, more effective approaches.

The majority of interventions that have attempted to increase children’s PA or reduce their SV have been delivered during curriculum time at school and have required manipulation of physical education provision, or involved educational components that are designed to increase children’s ability to change their behavior [11,12]. However, a number of systematic reviews and meta-analyses have shown that PA / SV interventions delivered within the school setting have reported null, weak or inconsistent effects [11,12]. Where interventions were successful, they tended to use several different intervention strategies [11]. As such, non-school approaches that utilize multiple intervention strategies need to be investigated [16].

Parents provide key socializing influences on their children’s PA [17] with parental support for PA being strongest before children’s transition to adolescence [18]. Parental PA facilitation (i.e., providing access, financial and transportation forms of PA support) is positively associated with objectively-assessed PA [18,19] among children. In addition, parental restriction of SV is associated with lower child SV [20]. Efforts to empower parents with the knowledge, skills, resources and support to facilitate active lifestyles among their children are warranted.

Although it has been argued that PA and SV are distinct behaviors [21] and that change in one behavior does not necessarily affect the other, it is important to recognize that reducing SV time can make more time in which it is possible to engage in PA. Reducing SV and increasing PA together also offers contextually compatible targets, which allow families to tailor behavior change interventions to their own requirements. Providing separate interventions for each would not be pragmatic in the public health context, whereas addressing both has the potential to increase effectiveness. There is, however, a shortage of research focusing on how parents can help their children engage in more PA and less SV. A systematic review of PA interventions found only four family-based studies that met the study inclusion criteria and as such there is insufficient evidence to draw conclusions on the utility of this approach [11]. As all four studies were conducted in the US and three focused exclusively on children from minority ethnic groups, the extent to which their findings are applicable to other countries and population groups may be limited. Furthermore, a review of multiple-component interventions to increase PA also highlights the absence of parent engagement in current approaches [22]. No study was found involving working directly with families, and parent engagement was restricted to newsletters home or the occasional parent evening. This lack of research is surprising as group-based parenting program interventions have been shown to be successful in engaging parents in providing support for behavior change in their children [23]. For example, Golan and colleagues have shown that interventions involving parents have been successful in childhood obesity treatment [24-28]. To date, no study has developed a parenting intervention to increase PA and reduce SV for non-obese children.

Behavior change interventions that have been based on psychological theory tend to be more successful than those that have not [29]. As well as improving impact, theory also supports evaluation design by assisting with the identification of key mediators of behavior change [29]. From an intervention perspective, self-determination theory (SDT) focuses on fostering autonomous (voluntary) types of motivation and feelings of competence, autonomy and belonging [30]. Previous research has found associations between these factors and self-reported exercise behavior and pedometer counts in children and adolescents [31,32] and as such, SDT could provide a useful basis for intervention. As SDT [30,33] addresses the role of social agents in fostering people’s motivation it may be particularly appropriate for working with parents to help their children feel more physically competent and build motivation in children for increased PA and reduced SV behaviors.

In light of the evidence reported above, we developed a new PA / SV group-based parenting intervention called Teamplay which incorporates best practice in group-based parent programs to deliver a SDT informed intervention. In this paper we report on the design of the Teamplay intervention and a feasibility trial evaluation of the intervention. The six specific aims of the feasibility trial were to:

1. Develop an intervention, an intervention manual, and resources for a group parenting program to promote increased PA and decreased SV in children.

2. Assess the feasibility of recruiting and retaining parents of 6–8 year old children to a PA/SV parenting program.

3. Examine the feasibility of collecting accelerometer data from children of this age and their parents.

4. Examine the potential change in MVPA and SV as a result of participating in the intervention and the potential future effect size.

5. Conduct post-intervention qualitative work to identify any factors that affect the measurement processes.

6. Provide the necessary information to calculate the sample size for an adequately powered RCT evaluation of the Teamplay intervention.

## Methods

A two arm individualized randomized controlled feasibility trial was used to evaluate the Teamplay intervention. Participants were parents with at least one child aged 6–8 years recruited from two neighboring wards in Bristol (UK). One ward was selected from the lowest and one from the middle tertiles of deprivation according to the index of multiple deprivation (IMD) [34] for the city of Bristol in order to sample approximate low and middle socioeconomic status areas of the city. The study was approved by a University of Bristol ethics committee. Participants were informed about the study in writing and in person, and written informed consent was obtained for all participants.

Participants were recruited via leaflets and advertisements that were distributed across the community in coffee shops, children’s centers, play groups, school playgrounds and community centers and face-to-face in schools and community centers. Based on our extensive formative research [35,36], the recruitment materials promoted a ‘Free 8-week course for parents’ which had the tag line “less stress, more fun in family life”. These materials focused on the parenting aspects of the course which many of the participants mentioned to the intervention staff as a key reason for joining the course. Moreover, to ensure that the course appealed to all parents, and not only parents of overweight or obese children the intervention materials focused on focused on having fun through physical activity and did not mention obesity or other health-related conditions. Parents were invited to meet the study team at an informal coffee morning at their child’s school or local community venue or to contact the study team by telephone. At these meetings parents were provided with information about the interventions and study procedures (including data collection and the randomization procedure). Parents were also asked to indicate if they would like to attend either a morning course (with free childcare) or an evening course (no childcare provided). Participants who consented to take part were randomized, within their chosen course preference to the intervention or control arm by an independent statistician with no other involvement in the study, using computer-generated random sequences.

### Description of intervention and control groups

Participants assigned to the intervention arm were invited to attend an eight week parenting program held in one of three local community centers. Parents attended without their children and each weekly session lasted two hours. The program was scheduled to coincide with school holiday periods with 4 weeks of content running before and after the primary school half term (a week long break in the middle of a school semester). To maximize opportunities for participants to attend, three programs were run, two during the day (for which a crèche was provided) and another during the evening. The control group received no additional input during the period of the intervention, but was provided with written materials summarizing the intervention content at the end of the study.

### Intervention development and delivery

The intervention content was informed by conducting in depth interviews with parents who reflected the intended user group [35] and an advisory group consisting of local council and parent-group representatives to help gather expert input and real life experiences which could be utilized in the intervention. The intervention was developed by the project team. As the intervention was focused on using the principles of SDT, we also drew on the SDT and educational experience of KRF and SJS. SDT helped to influence the development of material appropriate for children aged 6–8 years and informed the content on using active play to build autonomous motivation, confidence, and competence for increasing PA and reducing SV. The parenting aspects of the intervention were developed by project staff in consultation with members of Family Links, a UK parenting charity that trains professionals to run a parenting program (the Nurturing Program). Parenting aspects were aligned with SDT to encourage parents to use autonomy-supportive rather than controlling parenting strategies [33]. The content drew heavily on our formative research with parents which examined key issues that affected parental PA and SV behaviors, how these issues could be addressed and possible ways of structuring and delivering a group-based PA and SV parenting intervention [35,37].

A Teamplay leader manual was produced which gave detailed session plans for the 8-week course in order to ensure consistency of delivery across groups and the meeting of learning objectives. Each 2-hour session was made up of three main topic areas together with time for refreshments, games, parent feedback and the introduction of some tasks to be completed at home ('Put into Practice'). The program was delivered by two members of the research team (GFB and JKG) who had received Parent Group Leader training from Family Links. Material was delivered through group discussions and activities and used visual aids such as handouts, flip charts and display boards which were prepared in advance of the session. A final copy of the manual was produced incorporating the program leaders’ reflective learning gained in delivering the intervention and parent feedback on weekly content (Aim 1). The intended learning outcomes and an overview of content are presented for each of the 8 weeks in Table 1. Additional file 1: Table SA provides an example of full materials used to support delivery for week 2 of the intervention.

**Table 1:** Intended learning outcomes and content for the eight intervention sessions

**Week**	**Content**	**Intended learning outcomes**
**1**	Introduction to Teamplay	• Introduce parents to the Teamplay course and create a safe, enjoyable and respectful environment
	PA: What is it & why is it important?	• Help parents to identify/understand the benefits of PA
**2**	Physical activity recommendations	• Help parents to develop an understanding of what physical activity is.
	Active Play	• Introduce the value and importance of Active Play and support parents in identifying play ideas
	Praise	• Introduce praise and facilitating the learning of effective use of praise
**3**	Praise and criticism	• Help parents to recognize feelings surrounding praise and criticism and the impact upon behavior
	Screen-viewing (SV)	• Help parents look at SV in their household and identifying the pros and cons
		• Introduce strategies and tools to help parents to reduce SV
	Boundaries and consistency	• Introduce concepts of boundaries and consistency
**4**	Increasing ‘Inner motivation’	• Introduce parents to ‘Inner Motivation’, and top tips to encourage children’s inner motivation
	Family agreement	• Support parents to implement a family agreement for behaviors such as reducing SV
	Activity directory	• Help parents discover local activities that could contribute to their child’s PA
**5**	Appropriate expectations	• Understand the impact of expectations on self-esteem and behavior
	Helping children grow up	• Support parents in offering an empowering environment for their child
	Supporting children’s PA	• Support parents to discover ways to promote a fun and enjoyable PA environment
		• Introduce key movement skills for PA and provide practical ideas to support their development
		• Help parents support their child to experience success in PA through adapting activities
**6**	Personal power	• Help parents to use personal power to make healthy choices and support their child to do the same
	Self esteem	• Help parents understand the relationship between personal power, choices and self-esteem
		• Introduce the idea that PA can help improve self esteem
	Choices and consequences	• Introduce choices and consequences as a useful tool to reduce conflict
**7**	Communicating feelings	• Provide tools for parents and children to help communicate feelings
	Nurturing and downtime	• Create an understanding of the importance of looking after oneself
	Problem solving and negotiating	• Introduce problem solving and negotiation to help improve communication and reduce conflict
**8**	Summing up: useful tools	• Recap parenting tools and useful ways that they can be used
	Summing up: getting active	• Reaffirm the benefits and importance of PA and the ways in which it may be increased
	Continuing your journey	• Establish a family PA goal and signpost parents to more information and further support

### Baseline and follow-up measures

All measures were assessed at baseline (time 0) at the end of the intervention (time 1, week 8) and 2 months after the intervention had ended (time 2, week 16). The final assessment was designed to provide an indication of any long term effect of the intervention.

Parental employment status, parental ethnicity, parental gender and child age were assessed by parental report at baseline. Participant postcode was used to derive the Index of Multiple Deprivation (IMD) score for the home address. The IMD is an area level measure of deprivation that includes income, health, educational and employment status [34] with higher scores indicating higher levels of deprivation i.e. lower socioeconomic status (SES). Since the likely primary outcome of a full scale trial would be MVPA using accelerometer data, we assessed the feasibility of collecting accelerometer data (Aim 3) and the observed change in MVPA following participation in the intervention (Aim 4). All children and parents were asked to wear an Actigraph accelerometer (Model GT1M; ActiGraph LLC, FL, USA) for seven days at each time point. The Actigraphs were set to record data every 10 seconds. Periods of ≥60 minutes of zero values were defined as accelerometer “non-wear” time and discarded. Participants were included in the analysis if they provided ≥1 day of data with at least 480 minutes of data between 6 am and 11 pm. A cut-point of ≥2296 counts per minute [38] was used to identify mean minutes of weekday and weekend MVPA for the children. A cut-point of ≥2019 was used to categorize the parent activity data as MVPA [39].

Using a validated scale, parents were also asked to report the average number of hours per day that both they and the target child spent watching television. The assessment of TV viewing via a single question has been shown to correlate (r = 0.60) with 10 days of TV diaries among young children [40]. Parent and child TV viewing on weekday and weekend days at all three time points was categorized as < 2 hours per day, or ≥ 2 hours per day, corresponding to thresholds suggested by the American Academy of Pediatrics (AAP) TV viewing guidelines for children of 2 hours per day [41,42]. The UK does not have agreed SV thresholds [43].

### Attendance and post-study qualitative work

Attendance was recorded at each session (Aim 2). Intervention and control group participants who had initially provided consent to take part in an interview were randomly selected to be telephoned to see if they were still willing to take part. Parents were telephoned and interviews carried out until saturation had been met for intervention and control groups. A total of 16 interviews were conducted with intervention participants while 10 interviews were conducted with control participants. Interviews with intervention participants were conducted by a researcher who was independent of Teamplay. The interviews focused on thoughts about the data collection process, particularly how it could be improved and any factors that affected the wearing of the accelerometers. All interviews were recorded and transcribed.

### Analysis

#### Quantitative data

We used appropriate descriptive statistics (frequencies, percentages, means, standard deviations) to describe the recruitment and attendance data. Linear regression models were used to compare differences in means and 95% CI between the trial groups at follow up for the PA variables, adjusted for baseline PA. As the data are from a feasibility trial and we are not powered to detect a difference between groups, p-values are not reported. For descriptive purposes we also graphically presented the proportion of children and parents that met or exceeded the AAP TV viewing guidelines.

We reasoned that an intervention delivered to parents would have larger effects on behavior at weekends (i.e., when parents likely have more discretionary time to spend with children and facilitate their physical activity), would be observed at weekends, and indeed this was confirmed by the data. We therefore selected Weekend MVPA as our proposed primary outcome for a fully powered study. We calculated sample sizes needed to detect a mean difference of 10 minutes of weekend MVPA between intervention and control groups at the end of the intervention period. Sample size calculations are reported in Table 2. To provide a range of estimates of the likely sample size that would be needed for the trial different combinations of key parameters (i.e., type I and type II error levels) were used. Standard deviations of 12, 17 and 20 minutes were used as these three different values have been reported in the literature when describing the PA patterns of 6–8 year old children [44-46]. Finally the number of intervention and control programs that would need to be run for each set of sample size calculations were based on the assumption that there would be 10 participants recruited to each future intervention group. All analyses were performed using Stata 11 (Statacorp, College Station, Texas).

**Table 2:** Sample size calculations to detect a 10 minute difference in child weekend day moderate-to-vigorous physical activity between intervention and control groups

**SD (mins)**	**α**	**β**	**N required per group**	**Total N required for analysis**	**Total N inflated for attrition**	**Total N rounded and based on 10 participants per course group**	**Number of intervention groups**^**$**^	**Number of control groups**^**$**^
12	.80	.05	23	46	62.1	80	4	4
12	.80	.01	34	68	91.8	100	5	5
12	.90	.05	31	62	83.7	100	5	5
12	.90	.01	43	86	116.1	120	6	6
17	.80	.05	46	92	124.2	140	7	7
17	.80	.01	68	136	183.6	200	10	10
17	.90	.05	61	122	164.7	180	9	9
17	.90	.01	87	174	234.9	240	12	12
20	.80	.05	63	126	170.1	180	9	9
20	.80	.01	94	188	253.8	260	13	13
20	.90	.05	85	170	229.5	240	12	12
20	.90	.01	120	240	324	340	17	17

^$^ Assuming 10 participants per group.

#### Qualitative data

Analysis aimed at identifying factors that affected data completion rates, namely: 1) perceptions of the data collection process; 2) reasons for not wearing the accelerometers; and 3) strategies to improve data completion. Data were analyzed thematically to enable the exploration of accounts relating to these specific areas of interest. Transcripts were read and re-read by different members of the research team and a coding frame developed based on the themes identified. Using NVivo (Version 9, QSR, Southport, UK), meaningful content was coded and retrieved electronically, and then summarized to enable comparisons to be made within and across the interviews [47]. Quotes that were deemed to best represent the nature of each theme were then extracted.

## Results

### Quantitative data

There were 75 participants who provided consent and were randomized (Additional file 1: Figure SA). Twenty-seven participants withdrew post randomization. Of these, 7 intervention and 6 control group participants failed to attend or respond to contact attempts for baseline data collection, 6 participants withdrew once they’d been assigned to the control group, and 8 intervention group participants could not attend the course because of logistical issues (e.g. having an unwell child or a new baby). As a result, 25 participants allocated to the intervention group and 23 allocated to the control group provided baseline data. Some data were provided by 23 intervention and 15 control group participants at first follow up, and by 22 intervention and 11 control group participants at the second follow-up. Three intervention and 12 control group participants dropped out of the study during follow-up stages; 9 because of lost contact, 3 because they no longer wanted to take part in the study, and 3 because of personal factors (i.e. new job, bereavement and personal problems).

Descriptive statistics are presented for participants in Table 3. Control and intervention groups appear balanced on all variables at baseline except for parents’ weekend MVPA where intervention parents engaged in fewer minutes of MVPA than control parents (36.4 vs. 53.0 minutes per day). All but one of the adult participants were mothers. Mean weekday MVPA at baseline was 56 minutes for parents and 57 minutes for children, which is slightly less than the 60 minutes recommended for children aged 5–16 [43]. Around a third of the parents and children spent more than 2 hours per day watching TV at baseline. It is important to highlight that there was considerable variability in the employment, ethnicity and socio-economic position of the participants indicating that although there was a reasonable balance between arms and overall the sample was diverse.

**Table 3:** Descriptive characteristics of participants at baseline (time 0)

	**Intervention (n = 25)**	**Control (n = 23)**
	** n (%)**	** n (%)**
**Gender of study child**		
Female	8 (61.9)	11 (68.8)
Male	13 (38.1)	5 (31.3)
**Parent relationship to child**		
Mother	25 (100)	22 (95.7)
Father	0 (0)	1 (4.4)
**Ethnicity**		
White British	12 (48)	15 (65.2)
African	8 (32)	1 (4.3)
Indian	2 (8)	1 (4.3)
Caribbean	1 (4)	0
Any other White	0	4 (17.4)
Any other Asian	0	1 (4.3)
Any other ethnic group	1 (4)	0
Missing	1 (4)	1 (4.3)
**IMD**		
1^st^ Quartile (lowest IMD)	4 (16)	8 (34.5)
2^nd^ Quartile	8 (32)	4 (17.1)
3^rd^ Quartile	5 (20)	7 (30.4)
4^th^ Quartile (Highest IMD)	8 (32)	4 (17.1)
**Employment**	(n = 21)	(n = 19)
Not employed	9 (42.9)	10 (52.6)
1-11 (hours/week)	6 (28.6)	1 (5.3)
12-21 (hours/week)	3 (14.3)	2 (10.5)
21-36 (hours/week)	1 (4.8)	2 (10.5)
≥ 37 (hours/week)	2 (9.5)	4 (21.1)
**Child screen-viewing**		
**Weekday**		
<2 hours / day	16 (72.7)	13 (68.4)
≥2 hours / day	6 (27.3)	6 (31.6)
**Weekend**		
<2 hours / day	5 (23.8)	4 (21.1)
≥2 hours / day	16 (76.2)	15 (78.9)
**Parent screen-viewing**		
**Weekday**		
<2 hours / day	14 (66.7)	12 (63.2)
≥2 hours / day	7 (33.3)	7 (36.8)
**Weekend**		
<2 hours / day	8 (40.0)	8 (42.1)
≥2 hours / day	12 (60.0)	11 (57.9)
	**Mean (SD)**	**Mean (SD)**
Age of study child (n = 16)	6.6 (1.3)	8. (1.89)
Number of children	2.4 (0.9)	2.8 (1.1)
Mean IMD score	28.4 (16.4)	25.5 (17.7)
**Parent physic al activity**		
Weekday MVPA (mins / day)	56.63 (23.2)	56.14 (31.5)
Weekend MVPA (mins / day)	36.40 (15.5)	53.02 (44.3)
**Child physical activity**		
Weekday MVPA (mins / day)	57.29 (18.6)	57.427 (17.0)
Weekend MVPA (mins / day)	58.99 (28.7)	58.542 (41.1)

Weekly attendance ranged from 52% to 84% (Additional file 1: Figure SB). Two parents withdrew during the course of the intervention, but no parents who attended the course missed more than 4 sessions. The percentage of randomized participants who provided accelerometer data at Time 0, 1 and 2 is presented by trial arm in Additional file 1: Table SB. At Time 0, data were provided by 84% of intervention and 87% of control group children. At the first follow-up (Time 1) 56% of the intervention and 60% of the control children provided valid data with 68% of the intervention and control parents providing data. The number of valid days of accelerometer data per participant are presented by intervention arm and for the overall sample at each time point for children and adolescents in Additional file 1: Table SC. Overall there was a median of 4 days of valid data per child and 5 days per adult at each time point, but there was considerable variability between individuals.

The mean differences between the intervention and control children’s PA variables at Time 1 and Time 2 are presented in Table 4 after adjustment for baseline values and the household IMD score. The data in the table indicate that the intervention group engaged in 2.6 fewer minutes of weekday MVPA at Time 1 but 11 more minutes of weekend MVPA than the control group. Accelerometer counts per minute show a similar pattern. At Time 2 the intervention group engaged in 3 fewer minutes of MVPA on a weekday and 19 fewer minutes on a weekend day.

**Table 4:** Children’s physical activity data by trial arm and adjusted between group differences at time 1 (8 weeks) and 2 (16 weeks)

	**Intervention**		**Control**			
**Time 1**						
	**M**	**SD**	**M**	**SD**	**Adjusted difference in means (95% CI)†**	**N in comparison of means**
**MVPA/weekday (mins)**	60.44	21.69	63.93	17.43	−2.64 (−16.50 to 11.22)	28
**MVPA/weekend day (mins)**	77.58	23.65	68.53	30.93	11.04 (−7.87 to 29.94)	19
**CPM / weekday**	582.89	223.59	656.70	221.21	−89.34 (−257.85 to 79.16)	28
**CPM/ weekend day**	911.80	513.90	727.71	392.54	184.83 (−265.53 to 635.18)	14
**Time 2**						
**MVPA/weekday (mins)**	63.04	29.26	61.31	18.67	−3.23 (−22.35 to 15.86)	28
**MVPA/weekend day (mins)**	50.43	27.29	68.76	49.58	−19.65 (−56.57 to 17.27)	14
**CPM / weekday**	634.59	251.91	550.13	96.73	−32.56 (−177.59 to 122.47)	28
**CPM/ weekend day**	607.08	307.26	610.13	266.68	12.58 (−375.33 to 400.51)	14

*Note.* MVPA = moderate-to-vigorous physical activity.

† between group differences always compare to the intervention arm and are adjusted for baseline outcome scores & index of multiple deprivation.

The mean differences between the intervention and control parents’ PA variables at Time 1 and Time 2 are presented in Table 5 after adjustment for baseline values and the household IMD score. At Time 1 the intervention parents engaged in 9 more minutes of weekday MVPA and 13 more minutes of weekend MVPA. At Time 2 the intervention group engaged in 4 fewer minutes of weekday MVPA and 5 fewer minutes of weekday and weekend MVPA respectively.

**Table 5:** Parents’ physical activity data by trial arm and adjusted between group differences at time 1 (8 weeks) and 2 (16 weeks)

	**Intervention**		**Control**			
**Time 1**						
	**M**	**SD**	**M**	**SD**	**Adjusted difference in means (95% CI)†**	**N in comparison of means**
**MVPA/weekday (mins)**	68.01	24.52	58.00	36.69	9.06 (−7.54 to 25.66)	29
**MVPA/weekend day (mins)**	44.92	17.33	40.70	26.81	12.94 (−9.75 to 35.63)	19
**CPM / weekday**	481.28	135.04	424.90	217.05	25.57 (−66.64 to 117.79)	29
**CPM/ weekend day**	358.07	108.13	325.36	124.63	77.62 (−36.27 to 191.52)	19
**Time 2**						
**MVPA/weekday (mins)**	55.22	22.51	56.22	36.67	−3.65 (−20.27 to 12.97)	28
**MVPA/weekend day (mins)**	39.70	25.84	43.26	23.10	−4.92 (−35.36 to 25.51)	17
**CPM / weekday**	414.61	100.96	412.19	216.74	−28.60 (−120.28 to 63.09)	28
**CPM/ weekend day**	376.02	172.18	391.33	152.63	16.06 (−178.50 to 210.62)	17

*Note.* MVPA = moderate-to-vigorous physical activity, † between group differences always compare to the intervention arm and are adjusted for baseline outcome scores & index of multiple deprivation.

Please note that the n in the analyses varies due to incomplete data from some participants.

**Figure 1:**
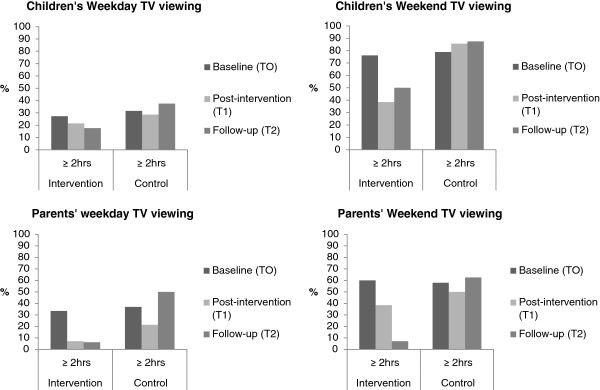
Proportion of children and parents watching > 2 hours of TV per weekday and weekend day by trial arm at all three time points.

The percentage of children and their parents spending more than 2 hours watching TV on a weekday and a weekend day is presented in Figure 1. The proportion of children spending more than 2 hours per day watching TV on weekdays decreased over time (t0 = 27% t1 = 21% t2 = 18%), while the control group values varied (32%, 29% and 37% respectively). The proportion of children in the intervention group watching ≥ 2 hours per day watching TV on weekend days decreased after the intervention (t0 = 76%, t1 = 39%, t2 = 50%), while the control group proportion increased slightly (79%, 86% and 87%). Parental weekday TV watching decreased from baseline in both groups, although more markedly in the intervention group who maintained this level at follow-up while control parents increased (intervention parents t0 = 33%, t1 = 7% and t2 = 6% watching ≥ 2 hours; control group parents t0 = 37%, t1 = 21%, t2 = 50).

Sample size calculations are reported in Table 2 using a range of scenarios regarding the standard deviation of child PA, alpha and beta levels. In all cases the required sample size for a trial was inflated by 35% to account for attrition and missing accelerometer data at Time 2 as observed in this study. A sample of between 80 and 340 participants, would be needed to detect a mean difference of 10-minutes of weekend MVPA. It is important to note, however, that a sample size of 240 participants (i.e. 12 intervention groups) would provide the ability to detect at a 10-minute difference with 80% power and at an alpha of 0.05 assuming a standard deviation of 20 minutes and would provide ample power for all other scenarios.

### Qualitative data summary

The mothers interviewed were happy with the principle that they and their child should wear the accelerometers. However, many mothers reported problems associated with wearing the accelerometers which deterred them from wearing them regularly or at all. Difficulty in remembering to wear the accelerometers was a common problem for both mothers and children.

“*I found it difficult at the beginning to remember to put it on … I suppose if I’d been going to the course I would have been more aware of it.” (Mother, control group)*

Some mothers for whom English was not their first language, reported that they did not understand the purpose of the accelerometer and did not realize that they had to wear it.

“I think at first, the last time you just, you explained to wear it but some people don’t understand it.” (Mother, intervention group)

“I couldn’t feel that I have to do it, it was like I just you can do it.” (Mother, intervention group)

Many mothers found wearing the accelerometers uncomfortable or annoying. This was particularly a problem when wearing clothing without belt loops to attach the accelerometer to, as the elastic belt holding the monitors was conspicuous and would ride up.

“You can’t wear it with a dress, well you look a bit daft and it kind of wriggles up a bit.” (Mother, control group)

Although some mothers reported that their child was happy or even proud and excited to wear an accelerometer, many mothers also reported that their child also disliked wearing them and some refused to wear them at all. Similar to parent views, children also found wearing the accelerometers uncomfortable, or felt self-conscious or susceptible to taunts from school peers, as they were usually the only child in their class wearing one.

“My son, yeah. He, he wore it and then he was complaining like it was too tight sometimes.” (Mother, intervention group)

“Yeah, and some children laughing at her…at school… sometimes children was pushing and say “what’s this?”… and they say “why you wearing this, what happened with you?”.” (Mother, intervention group)

The mothers talked about ways that made wearing the accelerometers easier and also ways in which data collection could be improved in the future to increase accelerometer wear time. These suggestions are summarized in Table 6.

**Table 6:** Parent (N = 26) reported problems associated with wearing accelerometers, their own strategies for overcoming these problems, and their suggestions to improve compliance in future interventions

**Parent reported problem**	**Successful strategies used by parents**	**Parents’ suggestions for future intervention**
Difficulty in remembering to wear the accelerometers	• Keeping the accelerometers in a visible place when they are taken off at night	• Allowing parents to opt in/out of a text reminder service was seen as a positive way to remind parents to wear the accelerometers
	• Making wearing the accelerometers a habit	
Lack of understanding about the accelerometers		• Spend more time explaining why parents are asked to wear the accelerometers
		• Having the information translated into parents’ native language
The accelerometers being uncomfortable or not practical to wear (for parents and children)	• Choosing to wear clothes with belt loops	
Children feeling targeted at school due to being only child wearing the accelerometer	• Support from teachers and school staff	• Providing information on the project/physical activity for the whole class
	• Wearing the accelerometer underneath the school uniform	• Asking the whole class to be involved in the project
Children refusing to wear the accelerometers	• Parents encouraging their child to wear the monitor	
	• Some children were naturally interested and proud to wear the accelerometer	
Increasing motivation to wear the accelerometer for parents and children: Provision of data feedback	• Many parents and children were interested in seeing the results from the accelerometers	• Promoting to parents at the initial data collection that they will get feedback on their data
	• Knowing if their child was getting enough PA compared to the recommendations, knowing if they’d increased PA over the 3 time points, and having a comparison of other people or the average were all of interest	

## Discussion

The data presented in this study have shown that it is possible to recruit parents to a PA / SV parenting course but a relatively high number of participants withdrew from the study during the study process. As noted in the flow-chart, 7 intervention and 6 control group participants failed to attend or respond to contact attempts for baseline data collection and 6 participants withdrew once they'd been assigned to the control group. Although, the six participants (8% of overall sample) withdrawing after being assigned to the control group is regrettable, overall there was reasonable acceptance of being randomized to intervention or control arms. It is important to highlight that there was considerable variability in the demographic and ethnic profile of the participants suggesting that sample was diverse and that the program potentially has broad appeal.

The data presented in this study indicate that the Teamplay course attendance levels were high. Overall data provision rates were reasonable, but there were some participants who failed to provide three valid days of accelerometer data. The parental interviews suggested that reasons for non-wear included a lack of understanding of why the monitors needed to be worn, forgetting to wear the monitors and their visibility and inconvenience. The parents suggested that these issues could be mitigated by providing text reminders to wear the monitors, spending more time explaining the purpose of the accelerometers and providing information to the participating child’s school on PA and the purpose of the accelerometer. Additionally, finding ways to improve the appeal of the monitors to the children so that they did not feel “daft (self-conscious)” when wearing them would likely increase wear time.

The analyses of the quantitative data suggested that the intervention children increased their weekend MVPA by 11 minutes more than the control group at the first follow-up with no marked difference in terms of weekday MVPA. These effects were not maintained at the second follow-up, and indeed intervention children were active less than control children at this point. The descriptive analyses also showed that a smaller proportion of the intervention children watched 2 or more hours of TV at the weekend than the control group at the first follow-up with less marked differences for weekday TV. These differences were maintained at the second follow-up. Thus, the intervention appears to have yielded an immediate positive effect on weekend MVPA and TV viewing but additional strategies will be needed to maintain these effects. Such strategies might include an internet-based maintenance program or a less intense group intervention to maintain behavior change once the original 8 sessions have been completed. For example, a less intense component could involve meeting once a month to monitor/discuss progress. The positive effects on weekend PA and SV suggest that the intervention worked best at the weekend. This is logical, since this is when 6–8 year old children are likely to spend more time with their parents. The findings suggest that parent input led to this change in child behavior. As noted in the introduction to this paper, the bulk of research to increase children’s PA has focused on school-based approaches [11]. These studies tend to focus on the development of PA on a weekday and largely ignore weekend and non-school day PA. The Teamplay intervention may therefore provide a solution for this neglected aspect of children’s PA and SV.

Parents in the intervention group engaged in 9 more minutes of weekday MVPA and 13 more minutes of weekend MVPA than the control group at the first follow-up but these effects were not maintained at the second follow-up. As with children, the intervention parents were also less likely to spend 2 or more hours per day watching TV than parents in the control group at both the first and second follow-up. Thus, the intervention appears to have had a beneficial effect on parent PA / SV behaviors. This finding is consistent with some of the early parent-focused obesity treatment work which suggested the parent-only interventions yielded positive effects on the adiposity and cardiovascular risk profile of both the target child and the parent [48].

This study suggests that achieving a mean difference of 10 minutes MVPA between intervention and control children following intervention is feasible. As the effect of increased PA on risk factors is likely to be curvilinear there is no agreed number of “extra minutes” that confer health benefits, but a mean increase of 10-minutes per day would likely have a beneficial effect on cardiometabolic risk factors. For example, analysis of the US National Health and Nutrition Examination Survey (NHANES) shows that young people with 14–33 mean minutes MVPA per day (the second quartile) were 35% less likely to be in the top quartile for waist circumference, 42% less likely to be in the top quartile for high non-HDL cholesterol and 50% less likely to be the top quartile for systolic blood pressure when compared to the participants with 0–13 mean minutes of MVPA (the lowest quartile)[49].

The sample size calculations suggest that between 80 and 340 parent–child dyads would need to be recruited to conduct an adequately powered evaluation of the intervention. The considerable variability between these numbers is a function of differences in the variance (standard deviations) that have been reported for the MVPA of 6 to 8 year old children. Assuming 10 parents are recruited to each course, this equates to running between 4 and 17 eight-week interventions with ample allowance for attrition. However, as noted above, 240 participants (i.e. 12 courses) would provide the ability to detect at a 10-minute difference with 80% power and an alpha of 0.05, assuming a standard deviation of 20 minutes, and would provide ample power for all other scenarios. As such, a sample of 240 participants would appear to present a conservative sample size and such a sample of 12 courses could be conducted within a larger city if a rolling recruitment program were implemented.

### Strengths and limitations

Statements about the effects of the intervention versus control group are tentative as this was a feasibility study and there was a lack of statistical power to detect group differences. However, the study design and analysis is consistent with the nature of feasibility studies and it is important that these preliminary studies are conducted and the findings disseminated prior to conducting larger trials [50,51]. The sample for this study was small and it appears that on average, the children and parents recruited had PA levels at baseline that were already close to achieving the national guidelines. This suggests the sample was slightly biased towards parents who were relatively active and as such, there is a need to replicate in a larger sample with a wider distribution of PA levels. It is also important to note that although there was considerable variability in socioeconomic position and ethnicity we do not have any information about household income or adult composition of households and so cannot interpret if these factors affected participation in the study. It is also important to highlight that we only assessed TV viewing in this study and as such we were not able to assess the effect on other forms of SV including multi-SV where multiple devices are watched at one time [52]. The second follow-up also occurred only 2 months after the intervention had ended and a longer-term follow-up might be required to assess potential impact beyond the intervention period. Only one father participated in the parenting program and as such the results reported here cannot be generalized to fathers. The interviews highlighted the difficulties parents had with wearing the accelerometers and strategies to address this issue should be addressed in future work.

## Conclusions

The Teamplay intervention represents a promising parenting program in an under-researched field. The intervention was acceptable to parents, and all elements of the study protocol were successfully completed. This feasibility trial demonstrates that a trial with a sample size of 240 families would likely demonstrate effectiveness of this intervention, and that a trial would be appropriate. Qualitative data suggest simple changes to the trial protocol which could result in more complete data collection and study engagement. This feasibility trial demonstrates that a trial with a sample size of 240 families would be appropriate.

## Competing interests

Professor Stewart-Brown is vice-Chair of Parenting UK (http://www.parentinguk.org/). No other authors have any conflicts of interest to declare.

## Authors’ contributions

The study was conceived by RJ, SJS, KMT, SSB, KRF and PJL who secured funding. The quantitative data were analyzed by SJS with the qualitative data analyzed by GFB, JKG and KMT. RJ produced the first draft of the paper with all other authors providing sections and critically reviewing the paper. All authors approved submission.

## Supplementary Material

Additional file 1: Table SA Intended learning outcomes and detailed content for week 2. **Table SB.** Percentage of randomized parents and children per trial arm who provided accelerometer data at Time 0, 1 and 2. **Table SC.** Number of valid days of accelerometer data provided by parents and study children*. **Figure SA.** Flowchart of participants through the study. **Figure SB.** Percent of participants (n = 25) attending intervention sessions by program week. Click here for file
