# Supplementary material for: Feasibility trial evaluation of a physical activity and screen-viewing course for parents of 6 to 8 year-old children: Teamplay
Source: Int J Behav Nutr Phys Act. 2013 Mar 4;10:31. doi: 10.1186/1479-5868-10-31 (PMC3598924; doi:10.1186/1479-5868-10-31)
Supplement: Additional file 1: Table SA — Intended learning outcomes and detailed content for week 2. Table SB. Percentage of randomized parents and children per trial arm who provided accelerometer data at Time 0, 1 and 2. Table SC. Number of valid days of accelerometer data provided by parents and study children*. Figure SA. Flowchart of participants through the study. Figure SB. Percent of participants (n = 25) attending intervention sessions by program week. [file 1479-5868-10-31-S1.doc]

**Additional file 1: Table SA:** Intended learning outcomes and detailed content for week 2

| **Content** | **Intended learning outcomes** |
| --- | --- |
| **2.1 Tea and Coffee** | n/a |
| **2.2 Parent’s feedback** | n/a; |
| - 1. **Game** | - Reinforce the importance of play and provide parents with games ideas they might like to play at home with their children - Allow parents to have fun, lighten the mood of the group and help people to relax |
|  |  |
| **2.4 Physical activity recommendations**   - Group activity: what counts as physical activity - Small steps - Recommendations - Group discussion | - Help parents to develop an understanding of what physical activity is, what counts and recap the benefits it has for their child - Help parents to assess their child’s physical activity |
| **2.5 Active Play**   - What is active play? - Game: Why is active play important? - Group activity: Barriers to active play - How parents can support their child play | - Introduce the value and importance of Active Play and support parents in finding play ideas - Help parents recognise what makes it difficult for their child to be physically active and family difficulties around physical activity - Help parents to discover what makes being active difficult for their child and in their family setting - Encourage parents to use strategies to overcome some of these barriers. |
|  |  |
| Tea and coffee break | n/a |
| **2.6 Praise**   - Introduction to praise - Praise role play - Steps in giving praise effectively - Being and doing activity - Ways we can praise/encourage children | - Introduce praise and facilitating the learning of effective use of praise - Support parents to feel confident in using praise with their children |
| **2.7 Put into practice** | - Encourage parents to try things out at home and bring back their experiences of what worked (if they want to), what didn’t work so well and what the effects were |
| **2.8 Week 2 evaluation forms** | n/a |
|  |  |

**Additional file 1: Table SB**: Percentage of randomised parents and children per trial arm who provided accelerometer data at Time 0, 1 and 2.

|  |  | **Intervention** | | | **Control** | | | **Total sample** | | |
| --- | --- | --- | --- | --- | --- | --- | --- | --- | --- | --- |
|  |  | *N* | *%* | 95% CI | *N* | *%* | 95% CI | *N* | *%* | 95% CI |
| **Study children** | | | | | | | | | | |
| Time 0 | Valid | 21 | 84 | .69 to .99 | 20 | 86.96 | .73 to 1.0 | 41 | 85.42 | .75 to.96 |
|  | Invalid | 4 | 16 | .10 to .31 | 2 | 8.70 | -.03 to .21 | 6 | 12.50 | .03 to .22 |
|  | Missing | - | - | - | 1 | 4.35 | -.04 to .13 | 1 | 2.08 | -.02 to .06 |
| Time 1 | Valid | 14 | 56.00 | .36 to .76 | 15 | 65.22 | .45 to .86 | 29 | 60.42 | .46 to .75 |
|  | Invalid | 8 | 32.00 | .13 to .51 | - | - | - | 8 | 16.67 | .06 to .28 |
|  | Missing | 3 | 12.00 | -.01 to .25 | 8 | 34.78 | .14 to .55 | 11 | 22.92 | .11 to.35 |
| Time 2 | Valid | 20 | 80.00 | .64 to .96 | 11 | 47.83 | .26 to .69 | 31 | 64.58 | .51 to.79 |
|  | Invalid | 3 | 12.00 | -.01 to .25 | 2 | 8.70 | -.03 to .12 | 5 | 10.42 | .01 to .19 |
|  | Missing | 2 | 8.00 | -.03 to .19 | 10 | 43.48 | .22 to .65 | 12 | 25.00 | .12 to.38 |
| **Parents** | | | | | | | | | | |
| Time 0 | Valid | 22 | 88 | .75 to 1.0 | 20 | 86.96 | .73 to 1.0 | 42 | 87.50 | .78 to .97 |
|  | Invalid | 3 | 12 | -.01 to .25 | 2 | 8.70 | -.03 to .21 | 5 | 10.42 | .02 to .19 |
|  | Missing | - | - | - | 1 | 4.35 | -.04 to .13 | 1 | 2.08 | -.02 to .06 |
| Time 1 | Valid | 17 | 68.00 | .49 to .87 | 12 | 52.17 | .31 to .74 | 29 | 60.42 | .46 to .75 |
|  | Invalid | 6 | 24.00 | .07 to .42 | 2 | 8.70 | -.03 to .21 | 8 | 16.67 | .06 to .28 |
|  | Missing | 2 | 8.00 | -.03 to .19 | 9 | 39.13 | .18 to .60 | 11 | 22.92 | .11 to .35 |
| Time 2 | Valid | 20 | 80.00 | .64 to .96 | 9 | 39.13 | .18 to .60 | 29 | 60.42 | .46 to .75 |
|  | Invalid | 3 | 12.00 | -.01 to .25 | 4 | 17.39 | .01 to .34 | 7 | 14.58 | .04 to .25 |
|  | Missing | 2 | 8.00 | -.03 to .19 | 10 | 43.48 | .22 to .65 | 12 | 25.00 | .12 to .38 |

*Note.* Valid = participant’s accelerometer data met the wear time criteria; Invalid = participant’s accelerometer data did not meet the wear time criteria; Missing = participant did not provide accelerometer data.

**Additional file 1: Table SC**: Number of valid days of accelerometer data provided by parents and study children*

|  |  | | | **Intervention** | | |  | | **Control** | | |  | | **Total sample** | | |
| --- | --- | --- | --- | --- | --- | --- | --- | --- | --- | --- | --- | --- | --- | --- | --- | --- |
|  |  | | | **Number of days** | | |  | | **Number of days** | | |  | | **Number of days** | | |
|  | *M* | | *SD* | | Median | Range | *M* | *SD* | | Median | Range | *M* | *SD* | | Median | Range |
|  | |  | | |  | Children | | | | | | | | | | |
| Time 0 | 3.2 | | 2.17 | | 4 | 0 to 6 | 4.41 | 2.11 | | 5 | 0 to 7 | 3.77 | 2.21 | | 4 | 0 to 7 |
| Time 1 | 2.77 | | 2.79 | | 2 | 0 to 7 | 4.60 | 1.80 | | 5 | 1 to 7 | 3.51 | 2.58 | | 4 | 0 to 7 |
| Time 2 | 3.52 | | 2.61 | | 4 | 0 to 7 | 4.54 | 2.82 | | 6 | 0 to 7 | 3.89 | 2.69 | | 4 | 0 to 7 |
|  | |  | | |  | Parents | | | | | | | | | | |
| Time 0 | 4.04 | | 2.21 | | 5 | 0 to 7 | 4.64 | 2.17 | | 5.5 | 0 to 7 | 4.32 | 2.19 | | 5 | 0 to 7 |
| Time 1 | 3.39 | | 2.71 | | 3 | 0 to 7 | 4.79 | 2.67 | | 5.5 | 0 to 7 | 3.92 | 2.74 | | 5 | 0 to 7 |
| Time 2 | 4.26 | | 2.61 | | 6 | 0 to 7 | 3.77 | 2.98 | | 4 | 0 to 7 | 4.08 | 2.72 | | 5.5 | 0 to 7 |

*This data is for all participants who wore the accelerometer (i.e were not classified as “missing” or invalid)

**Additional file 1: Figure SA: Flowchart of participants through the study**

**
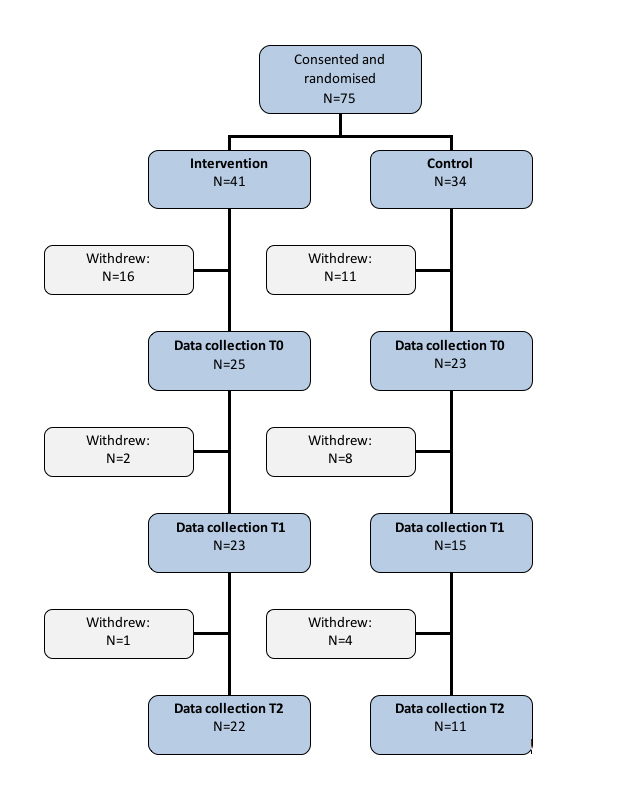
**

DC = Data Collection

TO = Time 1; T1 = Time 1 (end of intervention), T2 = Time 2 (8 weeks after end of intervention)

**Additional file 1: Figure SB: Percent of participants (n=25) attending intervention sessions by programme week**
